# Comparative Diagnostic Performance of IOTA Simple Rules, the ADNEX Model, and ACR O-RADS in a Selected Surgical Cohort of Adnexal Masses: A Retrospective Study

**DOI:** 10.3390/jcm15176717

**Published:** 2026-08-29

**Authors:** Lijun Wu, Yun Wu, Tao Hu, Juan Qian, Zhaoli Fan, Pingyang Zhang

**Affiliations:** 1 Department of Cardiovascular Ultrasound, Nanjing First Hospital, Nanjing Medical University, Nanjing 210006, China; wulijun.1@163.com; 2 Department of Ultrasound, Women’s Hospital of Nanjing Medical University (Nanjing Women and Children’s Healthcare Hospital), Nanjing 210004, China; nininunu26@163.com (Y.W.); hutao1979@126.com (T.H.); ctandqj@126.com (J.Q.); fzlyyan@163.com (Z.F.)

**Keywords:** adnexal mass, ultrasonography, IOTA simple rules, ADNEX model, ACR O-RADS, diagnostic performance

## Abstract

**Background/Objectives**: The accurate preoperative risk stratification of adnexal masses is clinically important. This study compared the diagnostic performance of the International Ovarian Tumor Analysis (IOTA) Simple Rules (SR), the Assessment of Different NEoplasias in the adneXa (ADNEX) model, and the American College of Radiology Ovarian-Adnexal Reporting and Data System (ACR O-RADS). **Methods**: This retrospective comparative study analyzed 429 women from a selected surgical dataset with pathology restrictions at a tertiary hospital in Nanjing, China, between January 2022 and October 2024. Two experienced gynecologic ultrasonographers independently applied SR, ADNEX (test-positive at a malignancy risk ≥ 10%), and O-RADS (test-positive at categories 4–5). Surgical histopathology served as the reference standard. Diagnostic performance was evaluated using exact binomial 95% confidence intervals (CIs), paired comparisons, receiver operating characteristic (ROC) analysis, ADNEX calibration, observed malignancy proportions across O-RADS categories, and interobserver agreement. **Results**: Among the 429 selected index adnexal masses, 114 (26.6%) were malignant. SR produced inconclusive classifications for 81 masses (18.9%). When SR-malignant and SR-inconclusive classifications were both regarded as positive indications for referral, SR achieved a sensitivity of 83.3% (95% CI, 75.2–89.7%) and a specificity of 84.1% (95% CI, 79.6–88.0%). ADNEX achieved a sensitivity of 86.0% (95% CI, 78.2–91.8%) and a specificity of 81.9% (95% CI, 77.2–86.0%), whereas O-RADS achieved a sensitivity of 89.5% (95% CI, 82.3–94.4%) and a specificity of 83.5% (95% CI, 78.9–87.4%). The AUCs for ADNEX and O-RADS were 0.92 and 0.90, respectively, with no statistically significant difference identified by paired DeLong analysis (*p* = 0.16). After Holm adjustment, none of the pairwise comparisons of sensitivity or specificity reached statistical significance. ADNEX exhibited apparent overall underprediction (calibration intercept, 0.865; calibration slope, 1.022; Brier score, 0.096). For O-RADS categories 2 and 4, the point estimates of observed malignancy proportions fell outside the corresponding predefined risk ranges. Interobserver agreement was almost perfect for O-RADS (*κ* = 0.89) and substantial for ADNEX (*κ* = 0.79) and SR (*κ* = 0.68). **Conclusions**: Within this selected surgical dataset subject to pathology-based restrictions, SR, ADNEX, and O-RADS demonstrated distinct diagnostic performance profiles. Following Holm adjustment, no statistically significant differences in sensitivity or specificity were detected among the three systems. Analyses of masses receiving an inconclusive SR classification were exploratory and therefore do not establish the validity of a sequential diagnostic pathway. Prospective multicenter investigations involving unselected populations are warranted before these findings can be generalized to broader clinical populations.

## 1. Introduction

Ovarian cancer continues to represent a substantial health burden among women in China, ranking as the ninth most common malignancy in 2022 [1] and remaining associated with considerable mortality. The reported 5-year overall survival rate is approximately 45%, decreasing from 90% in patients with stage I disease to 4% in those with stage IV disease [2]. These statistics highlight the clinical necessity for accurate and timely characterization of adnexal masses [3]. Transvaginal ultrasonography is the first-line imaging modality for the evaluation of adnexal masses [4]. To promote standardized risk assessment and communication, several ultrasound-based risk stratification systems have been developed [5].

The International Ovarian Tumor Analysis (IOTA) Simple Rules (SR), introduced in 2008, demonstrated high diagnostic accuracy (sensitivity, 93%; specificity, 90%) but produced inconclusive classifications in approximately 24% of cases [6]. To overcome this limitation, the IOTA group developed the Assessment of Different NEoplasias in the adneXa (ADNEX) model in 2014, which provides an individualized probability of malignancy and further categorizes malignant lesions into borderline tumors, stage I–IV ovarian cancer, and metastatic tumors [7]. In 2020, the American College of Radiology (ACR) introduced the Ovarian-Adnexal Reporting and Data System (O-RADS) for ultrasound, followed by an updated version in 2022 [8,9]. O-RADS offers a standardized framework for malignancy risk stratification accompanied by management recommendations. Accordingly, these systems differ in both the nature of their outputs and their intended clinical applications: ADNEX provides an individualized estimate of malignancy risk, whereas O-RADS classifies each mass into a predefined categorical risk stratum.

Direct head-to-head evaluation of SR, ADNEX, and O-RADS within the same population remains informative because comparisons between separate studies may be affected by differences in case mix and cannot account for the paired application of these systems to the same index adnexal mass [10]. Such evaluations should differentiate overall discrimination from paired comparisons of sensitivity and specificity at prespecified classification thresholds. Calibration likewise requires a system-specific framework: individual ADNEX risk estimates can be directly compared with observed outcomes, whereas O-RADS categories correspond to predefined risk ranges rather than unique patient-level predicted probabilities. Interobserver agreement is additionally relevant because reproducible classification is essential for reliable clinical implementation.

Accordingly, this retrospective comparative study assessed SR, the ADNEX model incorporating CA-125, and ACR O-RADS in a selected pathology-restricted surgical dataset from a Chinese tertiary hospital. The objectives were to characterize diagnostic performance according to prespecified binary classification rules; compare sensitivity and specificity through paired analyses; compare the discriminatory performance of ADNEX and O-RADS; evaluate the apparent calibration of individual ADNEX risk estimates; compare category-specific observed malignancy proportions against predefined O-RADS risk ranges; and assess interobserver agreement. Surgical histopathology was employed as the reference standard.

## 2. Materials and Methods

### 2.1. Study Population

This retrospective study analyzed a pathology-restricted historical surgical dataset derived from the electronic medical record database of a tertiary women and children’s hospital in Nanjing, China. The dataset comprised women who underwent surgery for an adnexal mass between January 2022 and October 2024. During the original database extraction, masses with postoperative diagnoses of mature cystic teratoma, endometriotic cyst, or broad-ligament leiomyoma were excluded before compilation of the available dataset. Consequently, the resulting cohort was neither consecutive nor unselected, and the denominator and diagnosis-specific counts of the prefiltered records could not be retrospectively reconstructed.

Eligible women were aged ≥18 years and had a nonphysiological adnexal mass evaluated by preoperative ultrasound, available serum CA-125 results, surgery with histopathological assessment within 120 days of the index ultrasound examination, and no history of recurrent ovarian malignancy. The 120-day interval was selected to minimize temporal discordance between ultrasound evaluation and histopathological assessment, consistent with previous IOTA research [11]. Exclusion criteria applied within the available dataset included pregnancy, age < 18 years, recurrent ovarian malignancy, unavailable preoperative CA-125 results, and archived ultrasound examinations that were not evaluable.

### 2.2. Data Collection and Reference Standard

Within the available dataset, two investigators (T.H. and Z.F.) verified eligibility and extracted age, menopausal status, serum CA-125 concentration (reference range, 0.0–35.0 U/mL), preoperative ultrasound images and video clips from the picture archiving and communication system, as well as final histopathological diagnoses. Discrepancies were resolved through discussion.

Three ultrasound-based risk stratification systems were assessed: IOTA SR, IOTA ADNEX model, and ACR O-RADS. Surgical histopathology served as the reference standard. Diagnoses were categorized as benign, borderline, or malignant according to World Health Organization criteria. For the primary diagnostic performance analyses, borderline tumors were classified as malignant, consistent with previous IOTA validation studies [12].

The study was approved by the Medical Ethics Committee of Nanjing Women and Children’s Healthcare Hospital (2024KY-132; 21 October 2024). Written informed consent was waived given the retrospective design. All clinical images were de-identified prior to inclusion.

### 2.3. Image Analysis

Transvaginal ultrasonography was the primary imaging modality. Transabdominal ultrasonography was added when transvaginal assessment was not feasible or did not permit adequate visualization of an adnexal mass. Examinations were performed using Mindray Resona R9 (C5-1, 1–5 MHz; C10-3V, 3–10 MHz; Mindray Medical International Limited, Shenzhen, China) or Philips EPIQ 7 (C5-1, 1–5 MHz; ML6-15, 6–15 MHz; Philips Healthcare, Andover, MA, USA) systems. Images and video clips were archived in the picture archiving and communication system.

Before assignment of the diagnostic systems, archived examinations were evaluated for adequacy. An examination was classified as non-evaluable when the stored images or video clips did not allow reliable assessment of one or more variables required for ap-plication of SR, ADNEX, and O-RADS. Sixteen examinations were excluded because of inadequate archived images or video clips (*n* = 5), missing color Doppler documentation (*n* = 4), incomplete visualization of the adnexal mass (*n* = 2), or Doppler settings that prevented reliable evaluation of vascularity (*n* = 5). These examinations were excluded before definitive classification and statistical analysis because consistent application of all three systems could not be ensured.

Two gynecologic ultrasound specialists (L.W. and J.Q.), each with more than 10 years of experience, independently reviewed the evaluable archived examinations while blinded to the final histopathological outcomes. For ADNEX risk estimation, age, menopausal status, and serum CA-125 concentration were available to the reviewers. Predefined sonographic features, including maximum mass diameter, internal echogenicity, contour, septations, papillary projections, acoustic shadowing, ascites, peritoneal nodules, and color Doppler vascularity, were recorded. SR classifications, individual ADNEX malignancy-risk estimates, and O-RADS categories were then assigned.

For women with bilateral adnexal masses, a single index adnexal mass was selected for analysis. The mass exhibiting the greatest morphological complexity was selected; when morphological complexity was comparable, the larger mass was selected. The selected index mass was matched to the final histopathological diagnosis according to laterality, based on the side documented on the preoperative ultrasound examination and the corresponding left- or right-sided surgical pathology specimen. No woman in the study cohort had more than one adnexal mass involving the same side.

### 2.4. Risk Stratification Systems

The compared risk stratification systems are described as follows:

(1) IOTA SR: The IOTA SR system consists of 10 predefined sonographic features, including five benign (B-rules) and five malignant (M-rules) predictors, as originally described by Timmerman et al. [6]. Lesions were categorized as inconclusive when either (1) both B-rules and M-rules were present simultaneously or (2) neither B-rules nor M-rules could be applied. Detailed SR criteria are available at https://www.iotagroup.org.

(2) IOTA ADNEX Model: The IOTA ADNEX model incorporates three clinical variables and six sonographic variables to estimate the absolute probability of malignancy and to distinguish benign from malignant adnexal masses across multiple tumor subtypes, as described by Van Calster et al. [7]. For clinical implementation, a publicly accessible web-based calculator is available at https://www.evidencio.com/models/show/946 (accessed on 1 May 2025).

(3) ACR O-RADS US: O-RADS classification was assigned according to the 2022 ACR O-RADS US update [9]. Each mass was categorized using the published O-RADS US lexicon and corresponding risk-stratification algorithm. The system comprises six categories: O-RADS 0, incomplete evaluation; O-RADS 1, normal ovary; O-RADS 2, almost certainly benign (<1% risk); O-RADS 3, low risk (1% to <10%); O-RADS 4, intermediate risk (10% to <50%); and O-RADS 5, high risk (≥50%) [9]. O-RADS categories 0 and 1 were not present in the final analytic cohort. For binary diagnostic-performance analyses, O-RADS categories 4–5 were classified as positive, whereas categories 2–3 were classified as negative.

### 2.5. Statistical Analyses

Continuous variables are presented as mean ± standard deviation (SD) or median (interquartile range [IQR]), as appropriate, and were compared using Student’s *t* test or Mann–Whitney U test. Categorical variables are presented as frequencies (percentages) and were compared using the chi-square test or Fisher’s exact test, as appropriate.

Diagnostic performance was evaluated using sensitivity, specificity, positive predictive value (PPV), and negative predictive value (NPV), with exact binomial (Clopper–Pearson) 95% confidence intervals (CIs). ADNEX risk ≥ 10% and O-RADS categories 4–5 were classified as test-positive. For SR, masses classified as SR-malignant or SR-inconclusive were considered positive in the primary referral analysis, whereas SR-benign masses were classified as negative. A secondary analysis was restricted to masses with definitive SR classifications.

Receiver operating characteristic curves were constructed for individual ADNEX risk estimates and ordinal O-RADS categories, and the corresponding areas under the curves (AUCs) were compared using the paired DeLong test. ROC analysis was not conducted for SR because its categories were not interpreted as an ordinal individual-risk scale. Because all three systems were applied to the same index adnexal mass for each woman, sensitivity and specificity were compared using two-sided exact McNemar tests within malignant and benign masses, respectively. Absolute paired differences and corresponding 95% CIs were estimated using paired bootstrap resampling with 5000 resamples. Holm adjustment was applied separately to the three pairwise sensitivity comparisons and the three pairwise specificity comparisons.

An exploratory subgroup analysis was conducted among women whose index adnexal mass received an SR-inconclusive classification. ADNEX at the 10% threshold and O-RADS categories 4–5 were evaluated descriptively using contingency tables and exact binomial 95% CIs. Interobserver agreement was evaluated using Cohen’s kappa: unweighted kappa for binary ADNEX classification and quadratic-weighted kappa for three-category SR classification and ordinal O-RADS categories 2–5. Kappa values were interpreted according to the Landis and Koch framework [13].

ADNEX calibration was evaluated by grouping individual predicted risks into 10 quantile-defined groups and calculating the mean predicted risk, observed malignancy proportion, and exact binomial 95% CIs within each group. The Brier score and apparent calibration intercept and slope were also calculated. Because O-RADS categories specify risk ranges rather than unique individual predicted probabilities, formal probability-calibration metrics were not calculated; instead, category-specific observed malignancy proportions were descriptively compared with the predefined O-RADS risk ranges. Statistical analyses were performed using Python version 3.8.3 and SPSS version 25.0 (IBM Corp., Armonk, NY, USA). Reporting adhered to STARD 2015 and TRIPOD. All statistical tests were two-sided; Holm-adjusted *p* values < 0.05 were considered statistically significant for paired comparisons, whereas *p* values < 0.05 were considered statistically significant for all other analyses.

## 3. Results

### 3.1. Patient and Adnexal Mass Characteristics

**Figure 1:**
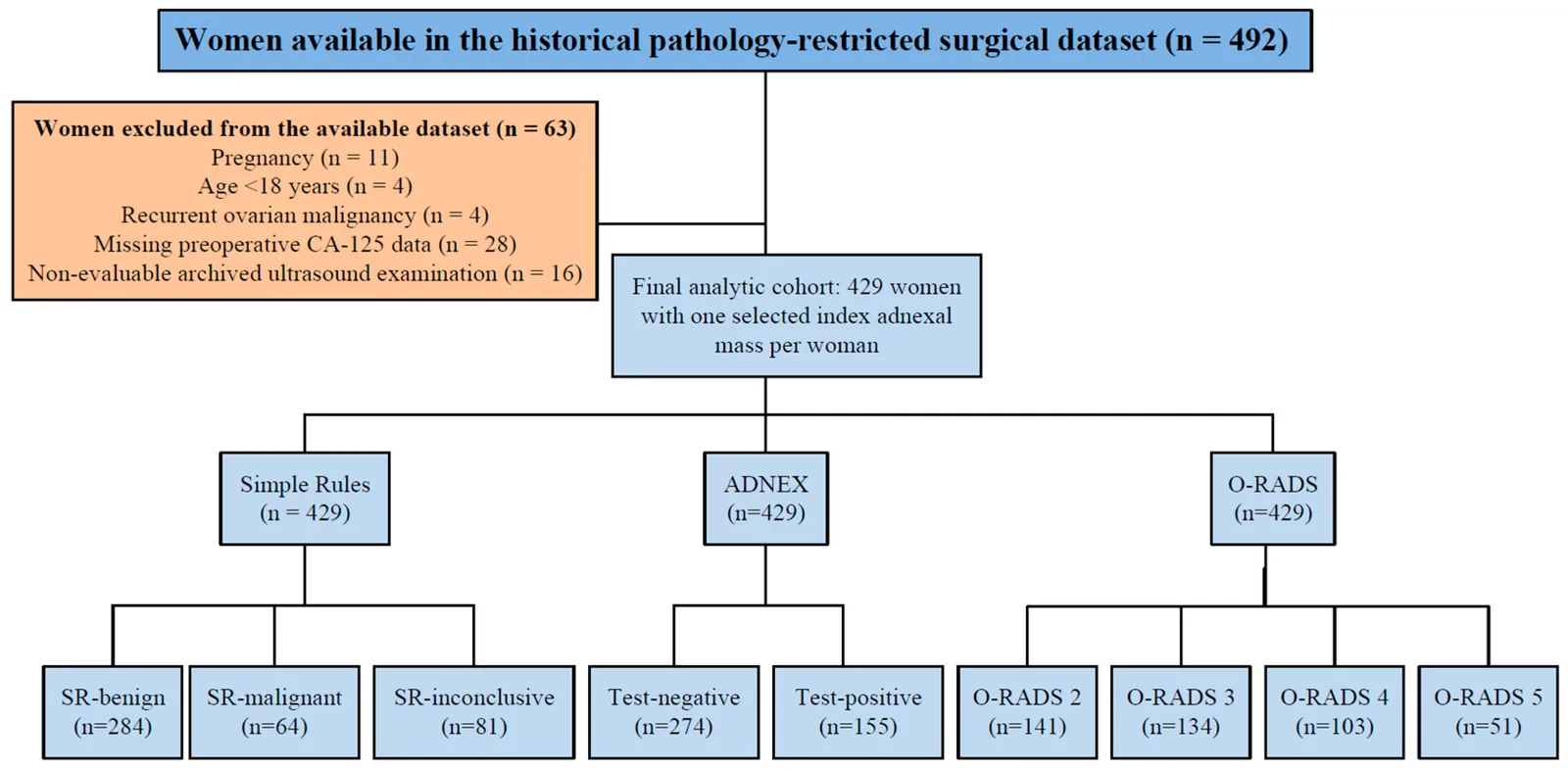
Flowchart of our study. Note: Data on the 492 women were derived from a historically pathology-restricted dataset and therefore did not constitute an unselected consecutive population. Before assembly of the available dataset, women with masses diagnosed postoperatively as mature cystic teratoma, endometriotic cyst, or broad-ligament leiomyoma were excluded; consequently, the denominator and diagnosis-specific counts of these prefiltered women could not be retrospectively reconstructed. Each woman included in the final analytic cohort contributed one selected index adnexal mass. Among the 16 non-evaluable archived ultrasound examinations, 5 had insufficient images or video clips, 4 lacked color Doppler documentation, 2 showed incomplete visualization of the adnexal mass, and 5 had Doppler settings that prevented reliable assessment of vascularity.

A total of 429 women, each with one selected index adnexal mass, were included in the final analysis (Figure 1). The mean patient age was 42.9 ± 14.9 years (range, 18–79 years); 290 (67.6%) were premenopausal and 139 (32.4%) were postmenopausal. The median interval from the index ultrasound examination to surgery was 9 days (IQR, 4–19 days). The median preoperative serum CA-125 concentration was 16.10 U/mL (IQR, 11.10–30.10 U/mL; range, 3.14–3875 U/mL). Of the 429 masses, 315 (73.4%) were benign and 114 (26.6%) were malignant, including 39 borderline tumors and 75 invasive carcinomas. Patients with malignant lesions were older and exhibited higher CA-125 concentrations than patients with benign lesions (both *p* < 0.05). Detailed patient characteristics and histopathological distribution are provided in Table 1 and Table S1.

### 3.2. Ultrasound Morphological Features

**Figure 2:**
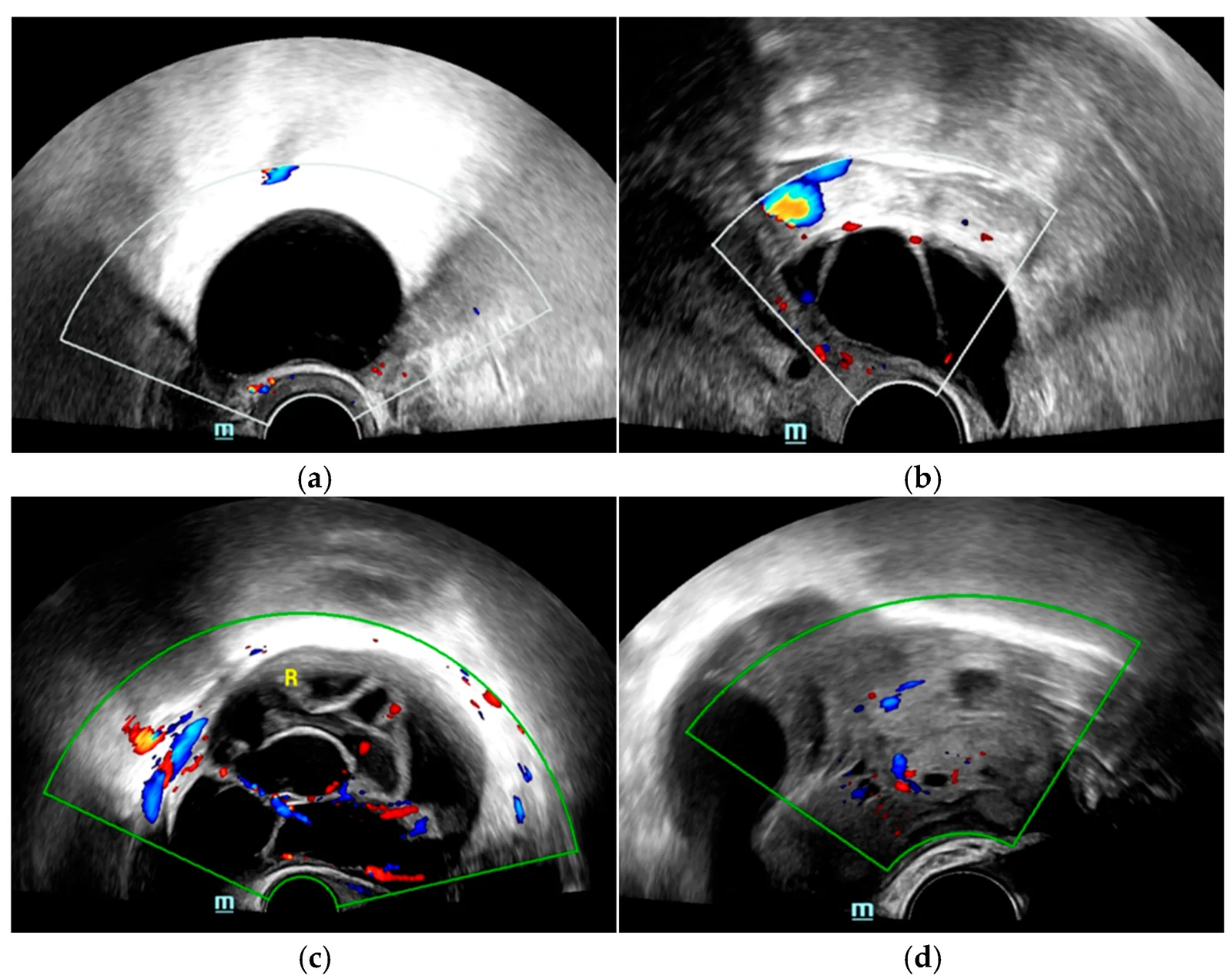
Representative cases demonstrating concordant and discordant classifications across IOTA SR, the ADNEX model, and ACR O-RADS. (**a**) A 53-year-old postmenopausal woman (CA-125 10.02 U/mL): a unilocular cystic right adnexal mass (5.92 × 4.87 cm; Color score 1), classified as benign by SR, with a 1.4% malignancy risk by ADNEX, and as O-RADS 2; histopathology: serous cystadenoma. (**b**) A 41-year-old premenopausal woman (CA-125 17.84 U/mL): a multilocular cystic left adnexal mass (6.74 × 5.11 cm; Color score 2), classified as benign by SR, with a 1.6% malignancy risk by ADNEX, and as O-RADS 3; histopathology: paraovarian cyst. (**c**) A 34-year-old premenopausal woman (CA-125 19.11 U/mL): a multilocular cystic right adnexal mass (13.92 × 9.39 cm; Color score 2–3), classified as inconclusive by SR, with a 7.6% malignancy risk by ADNEX, and as O-RADS 4; histopathology: mucinous cystadenoma. (**d**) A 58-year-old postmenopausal woman (CA-125 225.46 U/mL): a predominantly solid right adnexal mass (9.39 × 9.36 cm; Color score 2–3), classified as malignant by SR, with an 85.9% malignancy risk by ADNEX, and as O-RADS 5; histopathology: clear cell carcinoma.

Significant differences between benign and malignant masses were observed in lesion type, maximum lesion diameter, morphologic appearance, color Doppler flow score, and the presence of ascites (all *p* < 0.001; Table S2). Representative cases demonstrating concordant and discordant classifications across the diagnostic systems are presented in Figure 2.

### 3.3. Diagnostic Performance of IOTA Simple Rules

SR yielded definitive classifications for 348 of the 429 selected index adnexal masses (81.1%) and inconclusive classifications for 81 masses (18.9%). The observed malignancy proportions were 6.7% (19/284) among SR-benign masses, 92.2% (59/64) among SR-malignant masses, and 44.4% (36/81) among SR-inconclusive masses. When SR-malignant and SR-inconclusive masses were classified as test-positive for referral, sensitivity was 83.3% (95/114; 95% CI, 75.2–89.7%), specificity was 84.1% (265/315; 95% CI, 79.6–88.0%), PPV was 65.5% (95/145; 95% CI, 57.2–73.2%), and NPV was 93.3% (265/284; 95% CI, 89.7–95.9%) (Table 2).

In the secondary analysis, after exclusion of the 81 SR-inconclusive masses, the sensitivity was 75.6% (59/78; 95% CI, 64.6–84.7%), the specificity was 98.1% (265/270; 95% CI, 95.7–99.4%), the PPV was 92.2% (59/64; 95% CI, 82.7–97.4%), and the NPV was 93.3% (265/284; 95% CI, 89.7–95.9%).

### 3.4. Diagnostic Performance of the ADNEX Model

At the prespecified 10% risk threshold, ADNEX classified 274 selected index adnexal masses as test-negative and 155 as test-positive. Malignancy was present in 16 test-negative masses (5.8%) and 98 test-positive masses (63.2%). The sensitivity was 86.0% (98/114; 95% CI, 78.2–91.8%), the specificity was 81.9% (258/315; 95% CI, 77.2–86.0%), the PPV was 63.2% (98/155; 95% CI, 55.1–70.8%), and the NPV was 94.2% (258/274; 95% CI, 90.7–96.6%) (Table 2).

**Figure 3:**
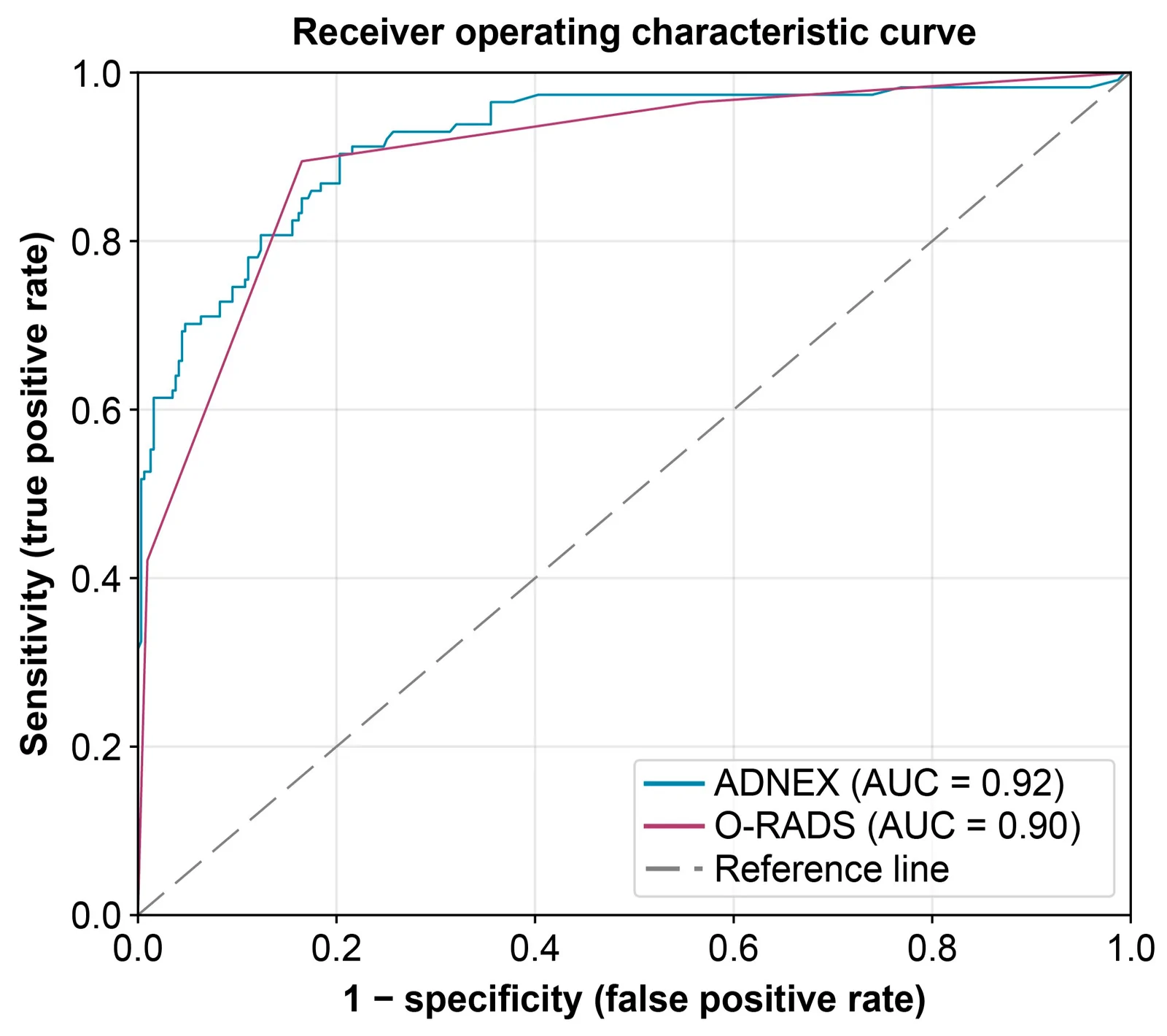
ROC curves for the diagnostic performance of the ADNEX model and O-RADS in discriminating between benign and malignant ovarian-adnexal masses (n = 429). ADNEX AUC = 0.92 (95% CI, 0.89–0.95). O-RADS AUC = 0.90 (95% CI, 0.87–0.94).

The AUC for individual ADNEX risk estimates was 0.92 (95% CI, 0.89–0.95) in the overall cohort (Figure 3), 0.90 (95% CI, 0.85–0.95) among premenopausal women, and 0.96 (95% CI, 0.93–0.99) among postmenopausal women.

### 3.5. Diagnostic Performance of ACR O-RADS

O-RADS assigned 275 selected index adnexal masses to categories 2–3 and 154 masses to categories 4–5. Category-specific observed malignancy proportions and corresponding exact binomial 95% confidence intervals are presented in Table 3. The observed malignancy proportions were 2.8%, 6.0%, 52.4%, and 94.1% for O-RADS categories 2, 3, 4, and 5, respectively. The point estimates for categories 2 and 4 fell outside their corresponding predefined risk ranges (<1% and 10% to <50%, respectively), although the associated CIs overlapped the relevant boundaries.

When O-RADS categories 4–5 were classified as test-positive, sensitivity was 89.5% (102/114; 95% CI, 82.3–94.4%), specificity was 83.5% (263/315; 95% CI, 78.9–87.4%), PPV was 66.2% (102/154; 95% CI, 58.2–73.6%), and NPV was 95.6% (263/275; 95% CI, 92.5–97.7%) (Table 2). The AUC for ordinal O-RADS categories was 0.90 (95% CI, 0.87–0.94) (Figure 3). Paired DeLong analysis showed no statistically significant difference in AUC between ADNEX and O-RADS (*z* = 1.41, *p* = 0.16).

### 3.6. Pairwise Comparisons of Diagnostic Performance

After Holm adjustment, none of the paired comparisons of sensitivity or specificity reached statistical significance (Table S3). O-RADS demonstrated a sensitivity 6.1 percentage points higher than that of SR (95% CI, 1.8–11.4 percentage points; adjusted *p* = 0.117). None of the remaining pairwise comparisons of sensitivity or specificity were statistically significant (all adjusted *p* ≥ 0.688).

### 3.7. Diagnostic Performance of ADNEX and ACR O-RADS in Women with SR-Inconclusive Masses

Among the 81 women with an SR-inconclusive index adnexal mass, 36 (44.4%) had malignant masses and 45 (55.6%) had benign masses. At the prespecified 10% threshold, ADNEX achieved a sensitivity of 83.3% (30/36; 95% CI, 67.2–93.6%) and a specificity of 60.0% (27/45; 95% CI, 44.3–74.3%). When O-RADS categories 4–5 were classified as positive, O-RADS achieved a sensitivity of 97.2% (35/36; 95% CI, 85.5–99.9%) and a specificity of 35.6% (16/45; 95% CI, 21.9–51.2%). ADNEX classified 6 malignant masses and 27 benign masses as negative, whereas O-RADS classified 1 malignant mass and 16 benign masses as negative. Additional diagnostic measures and complete contingency tables are presented in Table S4.

### 3.8. Calibration of the ADNEX Model

**Figure 4:**
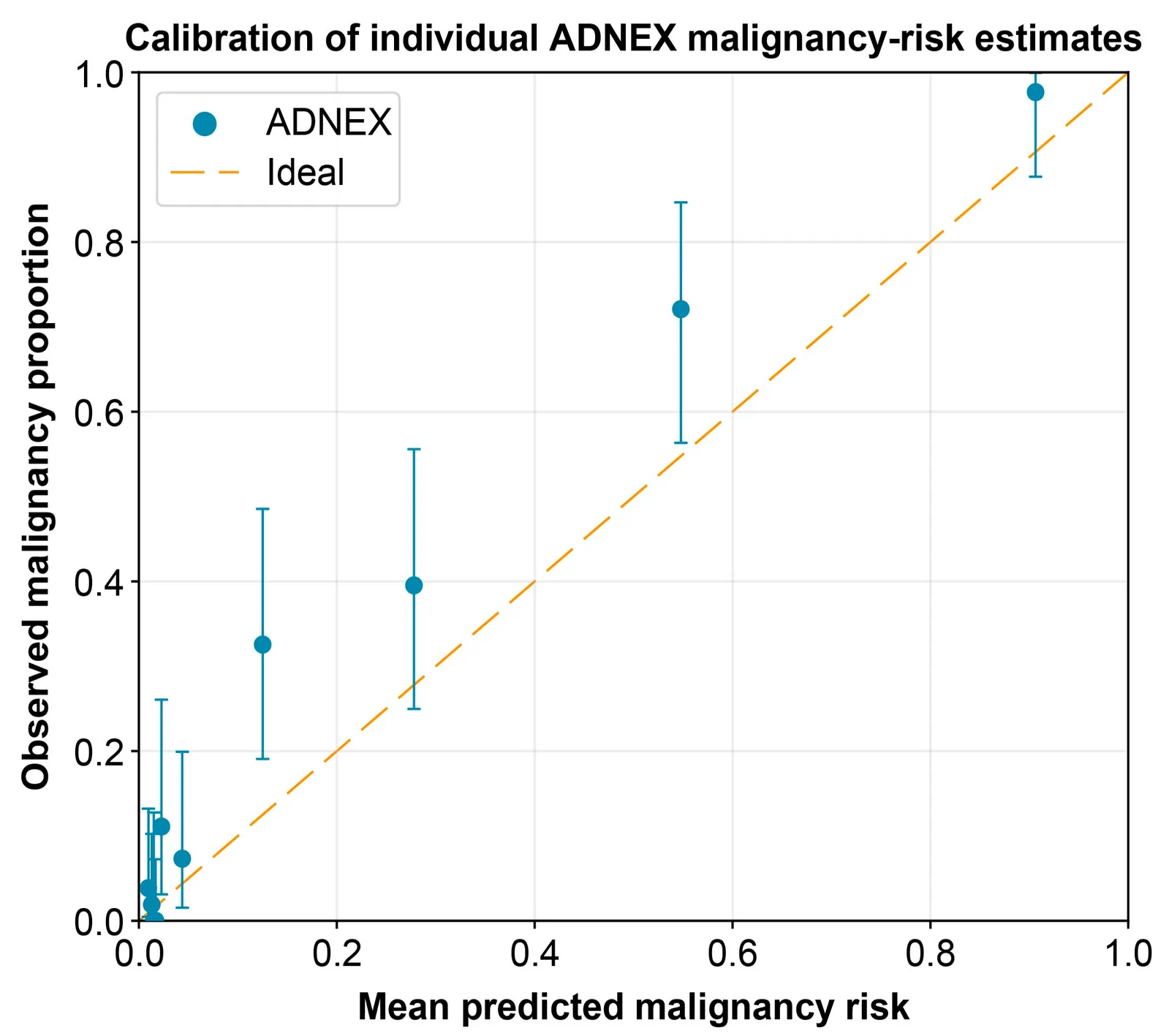
Apparent calibration of individual ADNEX malignancy-risk estimates. Each point represents one of 10 quantile-defined predicted-risk groups. The *x*-axis represents the mean predicted malignancy risk, whereas the *y*-axis represents the observed malignancy proportion. Error bars denote exact binomial (Clopper–Pearson) 95% confidence intervals. The dashed line indicates ideal calibration.

Calibration of the individual ADNEX malignancy-risk estimates is presented in Figure 4. Each point represents one of 10 quantile-defined predicted-risk groups. Across these groups, the observed malignancy proportion exceeded the mean predicted risk in most groups, particularly among groups with intermediate and higher predicted risks. In the lowest-risk groups, the observed malignancy proportions were based on a limited number of malignant masses and were associated with wide exact binomial confidence intervals. The apparent calibration intercept was 0.865, the apparent calibration slope was 1.022, and the Brier score was 0.096.

### 3.9. Interobserver Agreement

Interobserver reproducibility was almost perfect for O-RADS (weighted *κ* = 0.89; 95% CI, 0.85–0.93) and substantial for both binary ADNEX classification at the 10% threshold (*κ* = 0.79; 95% CI, 0.73–0.85) and three-category SR classification (weighted *κ* = 0.68; 95% CI, 0.61–0.75).

## 4. Discussion

This retrospective comparative study assessed IOTA Simple Rules (SR), the ADNEX model, and ACR O-RADS in a selected, pathology-restricted surgical dataset. The three systems demonstrated distinct diagnostic performance profiles, and none showed statistically significant superiority in the prespecified paired comparisons of sensitivity or specificity. O-RADS yielded the highest sensitivity point estimate (89.5%), whereas SR yielded the highest specificity point estimate (84.1%); however, none of the pairwise differences remained statistically significant after Holm adjustment. Similarly, paired DeLong analysis showed no statistically significant difference in discriminatory performance between ADNEX and O-RADS. These findings should not be interpreted as evidence of equivalence because this study was neither designed nor powered as an equivalence study.

SR provided definitive classifications for 81.1% of selected index adnexal masses, whereas 18.9% were classified as inconclusive, consistent with patterns reported in the original SR study and subsequent validation literature [6,14,15]. The two SR analyses demonstrate the implications of handling inconclusive classifications differently. Classifying both SR-malignant and SR-inconclusive masses as positive for referral resulted in a sensitivity of 83.3% and a specificity of 84.1%. By contrast, restricting the analysis to masses with definitive SR classifications increased specificity to 98.1% but decreased sensitivity. Accordingly, an inconclusive SR result should be interpreted as an unresolved classification requiring further evaluation rather than as evidence of malignancy.

The exploratory analysis of women with an SR-inconclusive index adnexal mass provides preliminary evidence regarding the performance of ADNEX and O-RADS in this diagnostically challenging subgroup. Previous comparisons of these two systems in overall cohorts have demonstrated generally comparable performance [16]; however, their relative performance specifically among SR-inconclusive lesions remains less extensively characterized. At the prespecified thresholds, ADNEX classified a greater number of benign masses as negative but simultaneously classified more malignant masses as negative than O-RADS. This pattern reflects differing sensitivity–specificity tradeoffs rather than established superiority of either diagnostic system. Given the limited subgroup size, wide confidence intervals, and absence of a prespecified paired comparison, these results should be interpreted as exploratory. They do not establish the validity of an SR-first sequential diagnostic pathway; prospective studies should prespecify the testing sequence, classification thresholds, and associated clinical actions before evaluating such an approach.

ADNEX generates individualized preoperative probabilities of malignancy and can therefore be assessed using probability-calibration measures [7,17]. In the present cohort, the positive apparent calibration intercept (0.865), together with observed malignancy proportions exceeding the mean predicted risks in most quantile-defined groups, suggested overall underprediction. The apparent calibration slope was 1.022, whereas the Brier score was 0.096. These estimates represent apparent in-sample performance because prediction and evaluation were conducted within the same selected, pathology-restricted cohort; consequently, they should not be interpreted as evidence of external validation. In addition, the limited number of malignant masses in several low-risk groups produced wide confidence intervals and reduced the precision of calibration assessment within that risk range.

O-RADS was assessed differently from ADNEX because its categories specify risk ranges rather than unique individual predicted probabilities [9,18]. Accordingly, formal probability-calibration metrics were not calculated for O-RADS. In the present cohort, the observed malignancy point estimates for categories 2 and 4 fell outside their nominal risk ranges, although the corresponding exact confidence intervals overlapped the relevant boundaries. These findings emphasize the importance of reporting category-specific observed malignancy proportions together with predefined O-RADS risk ranges rather than interpreting each category as a unique individual predicted probability. Previous O-RADS validation studies have similarly examined category-based risk stratification across other populations [19,20,21].

Interobserver agreement was almost perfect for O-RADS and substantial for ADNEX and SR, indicating that all three systems could be applied reproducibly by experienced gynecologic ultrasonographers when adequate morphologic and Doppler information was available. However, 16 archived examinations were considered non-evaluable because the stored images or video clips did not permit comparable assessment of variables required for application of all three systems. Exclusion of these examinations may have introduced selection bias and may have influenced the apparent performance of the systems differently, particularly for approaches requiring detailed morphologic characterization and Doppler evaluation.

### Limitations and Future Work

This study has several important limitations. First, it was a retrospective single-center analysis based on a selected, pathology-restricted surgical dataset. Before cohort assembly, women with masses diagnosed postoperatively as mature cystic teratoma, endometriotic cyst, or broad-ligament leiomyoma had been excluded during the historical database extraction; consequently, the denominator and diagnosis-specific counts of these prefiltered women could not be retrospectively reconstructed. Accordingly, the cohort was neither unselected nor consecutive and may therefore have limited generalizability to women undergoing routine evaluation for adnexal masses. Second, the requirement for available preoperative CA-125 results may have introduced selection bias, particularly in the ADNEX analysis. Third, the requirement for surgical histopathology as the reference standard may have enriched the cohort with masses selected for surgery and reduced representation of masses managed conservatively. Fourth, exclusion of the 16 non-evaluable archived ultrasound examinations may have introduced additional selection bias and may have influenced the apparent performance of the three systems differently. Finally, the SR-inconclusive subgroup was limited in size, and calibration was assessed within the same cohort in which predictions were evaluated; therefore, neither the subgroup findings nor the calibration estimates represent external validation.

Future prospective multicenter studies should enroll women before the final histopathological diagnosis is known, retain the full spectrum of adnexal masses, and prespecify the management of missing CA-125 data and non-evaluable ultrasound examinations. Such studies should specify the clinical action associated with each positive test result and determine whether a prespecified sequential diagnostic strategy improves referral decisions and subsequent patient management.

## 5. Conclusions

In this selected, pathology-restricted surgical dataset, IOTA Simple Rules, the ADNEX model, and ACR O-RADS demonstrated distinct diagnostic performance profiles. After Holm adjustment, no statistically significant differences in sensitivity or specificity were identified among the three systems, and findings among women with an SR-inconclusive index adnexal mass remained exploratory. Prospective multicenter studies involving unselected populations are required before these findings can be generalized or used to support implementation of a sequential diagnostic pathway.

## Figures and Tables

**Table 1:** Characteristics of the 429 Study Participants.

Characteristic	Benign (*n* = 315)	Malignant (*n* = 114)	*t*/*χ^2^*/*z* Value	*p*-Value
Age (y, x ± s)	41.6 ± 14.71	46.4 ± 15.23	−2.98	0.003
Menstrual status			9.53	0.002
Premenopausal	226	64		
Postmenopausal	89	50		
CA-125 [U/mL, m (Q1, Q3)]	14.7 (10.2–21.2)	33.8 (19.2–98.9)	−9.87	<0.001
Higher	21	65	132.43	<0.001
Normal	294	49		

**Table 2:** Diagnostic Performance of IOTA Simple Rules, the ADNEX Model, and ACR O-RADS in 429 Women with Selected Index Adnexal Masses.

Method	Groups (Women)	Sensitivity (%)	Specificity (%)	PPV (%)	NPV (%)
		(95%CI)			
Simple Rules (primary referral analysis)	All	83.3 (75.2–89.7)	84.1 (79.6–88.0)	65.5 (57.2–73.2)	93.3 (89.7–95.9)
	Pre-M	78.1 (66.5–86.7)	85.4 (80.2–89.5)	60.2 (49.4–70.2)	93.2 (89.0–95.9)
	Post-M	90.0 (78.2–96.0)	80.9 (71.4–87.8)	72.6 (60.4–82.2)	93.5 (85.6–97.3)
Simple Rules (definitive classifications only)	All	75.6 (65.0–83.9)	98.2 (95.8–99.2)	92.2 (82.9–96.8)	93.3 (89.7–95.8)
	Pre-M	64.1 (48.1–77.6)	99.0 (96.4–99.8)	92.6 (76.8–98.7)	93.2 (89.0–95.9)
	Post-M	87.2 (72.6–95.7)	96.0 (88.8–99.2)	91.9 (78.1–98.3)	93.5 (85.5–97.9)
ADNEX (risk threshold, 10%)	All	86.0 (78.2–91.8)	81.9 (77.2–86.0)	63.2 (55.1–70.8)	94.2 (90.7–96.6)
	Pre-M	76.6 (64.8–85.5)	86.3 (81.1–90.5)	61.3 (50.2–71.4)	92.9 (88.7–95.7)
	Post-M	98.0 (89.4–99.9)	70.8 (60.3–79.9)	65.3 (53.8–75.5)	98.4 (91.6–99.9)
O-RADS (categories 4–5 positive)	All	89.5 (82.3–94.4)	83.5 (78.9–87.4)	66.2 (58.2–73.6)	95.6 (92.5–97.7)
	Pre-M	84.4 (73.6–91.7)	86.7 (81.6–90.8)	64.3 (53.4–74.0)	95.1 (91.4–97.4)
	Post-M	96.0 (86.3–99.5)	75.3 (65.1–83.5)	68.6 (56.4–78.7)	97.1 (89.9–99.5)

Note: For the primary IOTA Simple Rules referral analysis, SR-malignant and SR-inconclusive masses were considered test-positive and SR-benign masses were considered test-negative. The definitive-classification analysis excluded the 81 SR-inconclusive masses. ADNEX risk ≥ 10% and ACR O-RADS categories 4–5 were considered positive. Each woman contributed one selected index adnexal mass. Pre-M = premenopausal women; post-M = postmenopausal women.

**Table 3:** Observed Malignancy Proportions by ACR O-RADS Category and Comparison with Predefined Risk Ranges.

O-RADS Category	Total Masses, *n*	Malignant Masses, *n*	Observed Malignancy Proportion	95% CI	Predefined O-RADS Risk Range
2	141	4	2.8%	0.8–7.1%	<1%
3	134	8	6.0%	2.6–11.4%	1% to <10%
4	103	54	52.4%	42.4–62.4%	10% to <50%
5	51	48	94.1%	83.8–98.8%	≥50%

## Data Availability

The data presented in this study are available on request from the corresponding author due to ongoing validation analyses targeting the lower-seniority (junior resident) group.

## Supplementary Materials

The following supporting information can be downloaded at https://www.mdpi.com/article/10.3390/jcm15176717/s1: Table S1: Final histopathological diagnoses of selected index adnexal masses; Table S2: Ultrasound morphological features of selected index adnexal masses according to final histopathology; Table S3: Paired comparisons of sensitivity and specificity among IOTA Simple Rules, ADNEX, and ACR O-RADS; Table S4: Diagnostic performance of ADNEX and O-RADS among 81 women with an SR-inconclusive index adnexal mass.

## Abbreviations

The following abbreviations are used in this manuscript:

IOTA	International Ovarian Tumor Analysis
ADNEX	Assessment of Different NEoplasias in the adneXa
ACR	American College of Radiology
O-RADS	Ovarian-Adnexal Reporting and Data System
SR	Simple Rules
CI	Confidence interval
AUC	Area under the curve
SD	Standard deviation
IQR	Interquartile range
ROC	Receiver-operating-characteristics
PPV	Positive predictive value
NPV	Negative predictive value
