# Supplementary material for: Comparative Diagnostic Performance of IOTA Simple Rules, the ADNEX Model, and ACR O-RADS in a Selected Surgical Cohort of Adnexal Masses: A Retrospective Study
Source: J Clin Med. 2026 Aug 29;15(17):6717. doi: 10.3390/jcm15176717 (PMC13566749; doi:10.3390/jcm15176717)
Supplement: Supplementary file 1 [file jcm-15-06717-s001.zip › jcm-4452068-supplementary.pdf]

Manuscript title: *Comparative Diagnostic Performance of IOTA Simple Rules, the ADNEX Model, and ACR O-RADS in a Selected Surgical Cohort of Adnexal Masses: A Retrospective Study*

Table S1. Final histopathological diagnoses of selected index adnexal masses

Pathologic diagnosis	Premenopausal women (n = 290)	Postmenopausal women (n = 139)	Total (n = 429)
**Benign**			
Serous cystadenoma	68	26	94
Mucinous cystadenoma	65	15	80
Seromucinous cystadenoma	14	2	16
Follicular membrane fibroadenoma	9	10	19
Fibroma	7	8	15
Cystadenofibroma	6	3	9
Adenofibroma	3	2	5
Brenner tumor	0	1	1
Sex cord stromal tumor	2	2	4
Hydrosalpinx	1	0	1
Sclerosing stromal tumor	1	0	1
Struma ovarii	0	1	1
Wolffian tumor	0	1	1
Mesosalpinx cyst	14	4	18
Simple cyst	24	7	31
Parovarian cyst	12	7	19
**Borderline tumor**			
Serous cystadenoma	18	3	21
Mucinous cystadenoma	10	5	15
Seromucinous cystadenoma	0	1	1
Brenner tumor	2	0	2
**Malignant**			
Serous carcinoma	11	27	38
Mucinous carcinoma	0	2	2
Adult granulosa cell tumor	6	3	9
Endometrioid adenocarcinoma	3	4	7
Clear cell carcinoma	5	5	10
Immature teratoma	3	0	3
Yolk sac tumor	1	0	1
Metastatic carcinoma	5	0	5

Table S2. Ultrasound morphological features of selected index adnexal masses according to final histopathology.

**Morphological characteristics**	**Benign (n = 315)**	**Malignant (n = 114)**	**χ^2 value**	***P* value**
**Major lesion category**			172.67	< 0.001
Unilocular	154	6		
Unilocular solid	11	25		
Multilocular	102	8		
Multilocular solid	10	19		
Solid or predominantly solid	38	56		
**Maximum diameter (D)**			48.28	< 0.001
D ≤ 3 cm	18	3		
3 cm < D ≤ 5 cm	71	19		
5 cm < D ≤ 10 cm	191	55		
D > 10 cm	35	37		
**Solid with irregular contour**	1	32	90.46	< 0.001
**No. of papillary projections**			54.17	< 0.001
0	292	75		
1–3	21	27		
> 3	2	12		
**Color score**			129.36	< 0.001
1	289	50		
2	24	34		
3	2	20		
4	0	10		
**Ascites**	1	15	38.47	< 0.001
**Acoustic shadowing present**	4	3	1.51	0.219

Table S3. Paired comparisons of sensitivity and specificity among IOTA Simple Rules, ADNEX, and ACR O-RADS.

Comparison	Outcome	No. of lesions	A correct/ B incorrect, n	A incorrect/ B correct, n	Discordant pairs, n	Difference (A-B), percentage points (95% CI)	Exact McNemar P value	Holm-adjusted P value
SR vs ADNEX	Sensitivity	114	7	10	17	−2.6 (−9.6 to 4.4)	0.629	0.688
SR vs ORADS	Sensitivity	114	1	8	9	−6.1 (−11.4 to −1.8)	0.039	0.117
ADNEX vs ORADS	Sensitivity	114	3	7	10	−3.5 (−8.8 to 1.8)	0.344	0.688
SR vs ADNEX	Specificity	315	35	28	63	2.2 (−2.9 to 7.3)	0.450	1.000
SR vs ORADS	Specificity	315	19	17	36	0.6 (−3.2 to 4.4)	0.868	1.000
ADNEX vs ORADS	Specificity	315	23	28	51	−1.6 (−6.3 to 2.9)	0.576	1.000

Note: Sensitivity was compared among malignant lesions (n = 114), and specificity was compared among benign lesions (n = 315). For each comparison, A and B correspond to the order shown in the Comparison column. "A correct/B incorrect" and "A incorrect/B correct" denote discordant pairs. Differences were calculated as A minus B; a negative value indicates a higher performance estimate for system B. Confidence intervals for paired differences were estimated using paired bootstrap resampling (5,000 resamples). Two-sided exact McNemar tests were used for P values. Holm adjustment was applied separately to the three sensitivity comparisons and the three specificity comparisons.

Table S4. Diagnostic performance of ADNEX and O-RADS among 81 women with an SR-inconclusive index adnexal mass.

Model	True positive, n	False negative, n	True negative, n	False positive, n	Sensitivity (95% CI)	Specificity (95% CI)	PPV (95% CI)	NPV (95% CI)
ADNEX (≥10 % risk)	30	6	27	18	83.3% (67.2%–93.6%)	60.0% (44.3%–74.3%)	62.5% (47.4% – 76.0%)	81.8% (64.5% – 93.0%)
O-RADS (4-5)	35	1	16	29	97.2% (85.5%–99.9%)	35.6% (21.9%–51.2%)	54.7% (41.7% – 67.2%)	94.1% (71.3% – 99.9%)

Note: Of the 81 SR-inconclusive lesions, 36 were malignant and 45 were benign. For ADNEX, a malignancy risk ≥10% was considered test-positive. For O-RADS, categories 4–5 were considered test-positive. Exact binomial (Clopper–Pearson) 95% confidence intervals are reported. PPV: positive predictive value; NPV: negative predictive value.
